# Status and influencing factors of knowledge, attitudes, and practices regarding oxygen therapy among clinical nurses in Anhui Province, China

**DOI:** 10.3389/fpubh.2026.1945141

**Published:** 2026-09-28

**Authors:** Ling-li Jin, Bo Li, Rui-xiang Sun, Li-ping Yuan

**Affiliations:** Department of Yijishan Hospital, Wannan Medical College, Wuhu, China

**Keywords:** clinical healthcare workers, influencing factors, knowledge, attitudes, and practices, nursing management, oxygen therapy

## Abstract

**Background:**

To investigate the current status of knowledge, attitudes, and practices regarding oxygen therapy among clinical nurses in Anhui Province (with a predominantly nursing sample), analyze the associated factors, and provide a basis for developing targeted training strategies.

**Methods:**

Using convenience sampling, 1,172 clinical nurses from 24 hospitals in Anhui Province were selected from August to December 2025. A self-developed “Knowledge, Attitudes, and Practices Survey on Oxygen Therapy for Clinical Nurses” was used, and associated factors were analyzed using multiple linear regression.

**Results:**

The standardized scores for nurses’ knowledge, attitudes, and self-reported practices were (75.28 ± 9.03), (85.77 ± 11.71), and (79.99 ± 14.82), respectively. Multiple linear regression showed that gender, occupation, professional title, and hospital level were associated with the knowledge dimension (*p* < 0.05); occupation, education level, hospital level, and participation in oxygen therapy training were associated with the attitude dimension (*p* < 0.05); and attitude, professional title, years of work, and participation in training were associated with the practice dimension (*p* < 0.05). However, the physician sample was extremely small (*n* = 9), and the occupation-related finding should be interpreted with caution.

**Conclusion:**

The overall KAP levels are moderate, with positive attitudes but significant knowledge gaps. Training participation was associated with better attitudes and practices but not with knowledge scores, suggesting that current training may need to emphasize updated guidelines and pathophysiology. Given the predominantly nursing sample (99.2%), findings are most directly applicable to nursing staff.

## 1 Background

Oxygen therapy is an important treatment method for correcting hypoxemia by inhaling gas with an oxygen concentration higher than that of air, thereby increasing arterial blood oxygen partial pressure (PaO_2_), blood oxygen saturation (SpO_2_), and oxygen content in the blood. However, oxygen therapy is not completely harmless. Studies have shown that prolonged exposure to high concentrations of oxygen can cause multi-organ damage, involving mechanisms such as excessive generation of oxygen free radicals, cerebral vasoconstriction, and reduced tissue perfusion, which in turn increase the risk of necrosis in cardiac and brain cells (1, 2). In patients with COPD, inappropriately high oxygen concentration may not only aggravate hypercapnia and respiratory acidosis by inhibiting hypoxic respiratory drive, but also increase ventilation/blood flow imbalance by reversing hypoxic pulmonary vasoconstriction, and reduce CO_2_ transport capacity due to Haldane effect. Together, this leads to increased CO_2_ retention (3). Although oxygen therapy is widely used in clinical practice, its standardized implementation still faces challenges. Surveys have shown that clinical oxygen therapy has problems such as unclear target settings, insufficient monitoring, and arbitrary flow adjustments, basically remaining in a “ruleless” state (4). This non-standard oxygen therapy not only affects treatment efficacy but may also cause iatrogenic injuries such as hyperoxia (5). Domestic research has mostly focused on the rationality and standardization of oxygen therapy prescriptions. Systematic surveys on the knowledge, attitude, and practice (KAP) of clinical healthcare personnel regarding oxygen therapy are relatively scarce (6). Based on this, this study uses the knowledge-attitude-practice framework to investigate the current status of KAP regarding oxygen therapy among clinical healthcare personnel in Anhui Province and analyze its influencing factors, aiming to provide empirical evidence for constructing a hierarchical training system and formulating quality control standards for oxygen therapy.

## 2 Materials and methods

### 2.1 General information

Using a convenience sampling method, clinical medical staff from 24 hospitals in Anhui Province were selected as research subjects from August to December 2025. Inclusion criteria: ① holding a nurse practitioner certificate or physician practitioner certificate; ② engaged in clinical work for at least 1 year; ③ provision of electronic informed consent and voluntary participation. Exclusion criteria: ① trainees or interns; ② those not on duty during the survey period due to leave, external training, or other reasons. A total of 1,172 research subjects were ultimately included.

### 2.2 Methods

#### 2.2.1 General information questionnaire

Self-designed, including gender, age, education level, years of work experience, professional title, department, hospital level, employment type, source of oxygen therapy knowledge, whether oxygen therapy training has been received, etc.

#### 2.2.2 Clinical nurses’ knowledge, attitude, and practice survey on adult oxygen therapy

Based on the theoretical foundation of ‘knowledge, attitude, and practice,’ the research team systematically searched domestic and international oxygen therapy guidelines (7–9), related literature (10), and oxygen therapy group standards, and formed the initial draft of the questionnaire through group discussion. Six medical experts from the Department of Respiratory Medicine/Department of Critical Care Medicine/Department of Hyperbaric Oxygen and nine clinical nursing experts were invited to conduct two rounds of Delphi consultation. Based on the experts’ opinions, the items were revised to form the final version of the questionnaire. The final questionnaire demonstrated good psychometric properties: Scale-level Content Validity Index (S-CVI) was 0.92, with item-level CVI ranging from 0.78 to 1.00. Internal consistency was satisfactory (Cronbach’s *α* = 0.87 for the total scale; 0.79 for knowledge, 0.84 for attitude, and 0.88 for practice dimensions). Test–retest reliability over a 2-week interval (*n* = 30) yielded an intraclass correlation coefficient of 0.85. Exploratory factor analysis confirmed a three-factor structure explaining 58.7% of the total variance (KMO = 0.872, Bartlett’s test of sphericity *p* < 0.001). The questionnaire includes three dimensions: ① Knowledge dimension, 15 items, all single-choice questions, 5 points for a correct answer, 0 points for an incorrect answer, with a full score of 75; ② Attitude dimension, 6 items, using a 5-point Likert scale (1 = strongly disagree, 5 = strongly agree), with a full score of 30; ③ Practice dimension, 10 items, using a 5-point Likert scale (1 = never, 5 = always), with a full score of 50. The total score ranges from 0 to 155. For comparison purposes, the scores of each dimension are converted into standardized scores: Standard Score = (Actual Score/Total Score of the Dimension) × 100. Standardized scores ≥85 were defined as high level, 60–84 as medium level, and <60 as low level. This classification criterion is mainly based on the consideration of comparability between studies, and the cut-off value of clinical significance has not been rigorously verified externally. Therefore, we also pay attention to the results of continuous scores in the analysis (11).

### 2.3 Survey method

Electronic questionnaires were distributed using the Wenjuanxing platform. Each question was set as mandatory, with a response time limit of ≥3 min, and each device could only submit once. Nursing/medical affairs officers from each hospital were designated as liaisons, responsible for questionnaire distribution and quality control of collection. The platform was open for 4 months, collecting a total of 1,219 questionnaires. After excluding 47 invalid questionnaires with identical answers or internal logical inconsistencies, there were 1,172 valid questionnaires, resulting in an effective response rate of 96.14%.

### 2.4 Statistical analysis

Data analysis was performed using SPSS 25.0 software. Measurement data were described as mean ± standard deviation ( $\bar{x}\pm s$ ), and comparisons between groups were conducted using independent sample *t*-tests or one-way analysis of variance. Count data were described by frequency and composition ratio (%). Influencing factors were analyzed using multiple linear stepwise regression (Stepwise method). *p* < 0.05 was considered statistically significant. To ensure robustness of the regression results, we also performed a sensitivity analysis using dummy-coded categorical variables for education, professional title, and hospital level instead of continuous numerical coding. The direction and significance of the main findings were consistent with the primary analysis (data not shown in tables). For rigor, the dummy-coded results are considered exploratory, and future studies may use this approach to obtain more detailed between-group comparisons.

## 3 Results

### 3.1 Scores of healthcare workers’ knowledge, attitudes, and practices regarding oxygen therapy

The scores of healthcare workers’ knowledge, attitudes, and practices regarding oxygen therapy, as well as the top three and bottom three item scores in each dimension, are shown in Tables 1, 2, respectively (Figure 1).

**Table 1:** Scores of Medical Staff on knowledge, attitude, and practice of oxygen therapy.

Project	Actual score ( $\bar{x}\pm s$ )	Standardized score ( $\bar{x}\pm s$ )	Level distribution [number of people (*n*%)]		
			High	Middle	Low
Knowledge	56.46 ± 6.77	75.28 ± 9.03	122 (10.41%)	978 (83.45%)	72 (6.14%)
Attitude	25.73 ± 3.51	85.77 ± 11.71	613 (52.30%)	541 (46.16%)	18 (1.54%)
Practice	39.99 ± 7.41	79.99 ± 14.82	430 (36.69%)	639 (54.52%)	103 (8.79%)

**Table 2:** Scores of the top 3 and bottom 3 items of medical staff’s knowledge, attitude, and practice regarding oxygen therapy.

Project	Sort	Entry	Score
Knowledge	Top 1	2. Indications for oxygen therapy include	4.86 ± 0.85
	Top 2	15. Contents to Observe and Monitor During Oxygen Therapy	4.71 ± 0.91
	Top 3	13. Description of nasal high-flow oxygen therapy (HFNC)	4.68 ± 0.89
	Bottom 3	14. Description of oxygen therapy humidification (reverse scoring: correct answer is ‘not always required’)	3.23 ± 1.35
	Bottom 2	5. Description of preparations before administering oxygen therapy to patients (reverse scoring: correct answer is ‘not all items listed are routine prerequisites’)	2.73 ± 1.92
	Bottom 1	4. Which of the following is not a clinical manifestation of oxygen toxicity?	1.54 ± 1.16
Attitude	Top 1	1. Oxygen therapy plays an important role in the treatment of critically ill patients	4.57 ± 0.76
	Top 2	3. It is necessary to establish clear individualized oxygen therapy goals for patients receiving oxygen therapy.	4.42 ± 0.71
	Top 3	4. It is necessary to precisely regulate the inhaled oxygen flow and concentration according to the oxygen therapy goals.	4.42 ± 0.68
	Bottom 3	6. One should take the initiative to learn knowledge related to clinical oxygen therapy.	4.34 ± 0.68
	Bottom 2	5. Interested in clinical oxygen therapy knowledge	4.22 ± 0.72
	Bottom 1	2. Oxygen is a medicine	3.76 ± 1.22
Practice	Top 1	2. In daily work, try to avoid patients experiencing hypoxia.	4.29 ± 0.84
	Top 2	3. In daily work, try to avoid patients experiencing hyperoxia.	4.09 ± 0.94
	Top 3	5. Precisely control the inhaled oxygen flow rate and concentration according to oxygen therapy targets	4.05 ± 0.97
	Bottom 3	1. Consider the side effects of oxygen therapy during its administration to patients	3.95 ± 1.01
	Bottom 2	10. Actively accumulate knowledge related to clinical oxygen therapy	3.91 ± 0.99
	Bottom 1	7. For those at high risk of CO_2_ retention, avoid SpO_2_ reaching 100%	3.62 ± 1.16

Knowledge items marked as ‘reverse scoring’ are negatively worded items for which the correct response is the opposite of the literal statement; these items were recoded accordingly during scoring.

**Figure 1:**
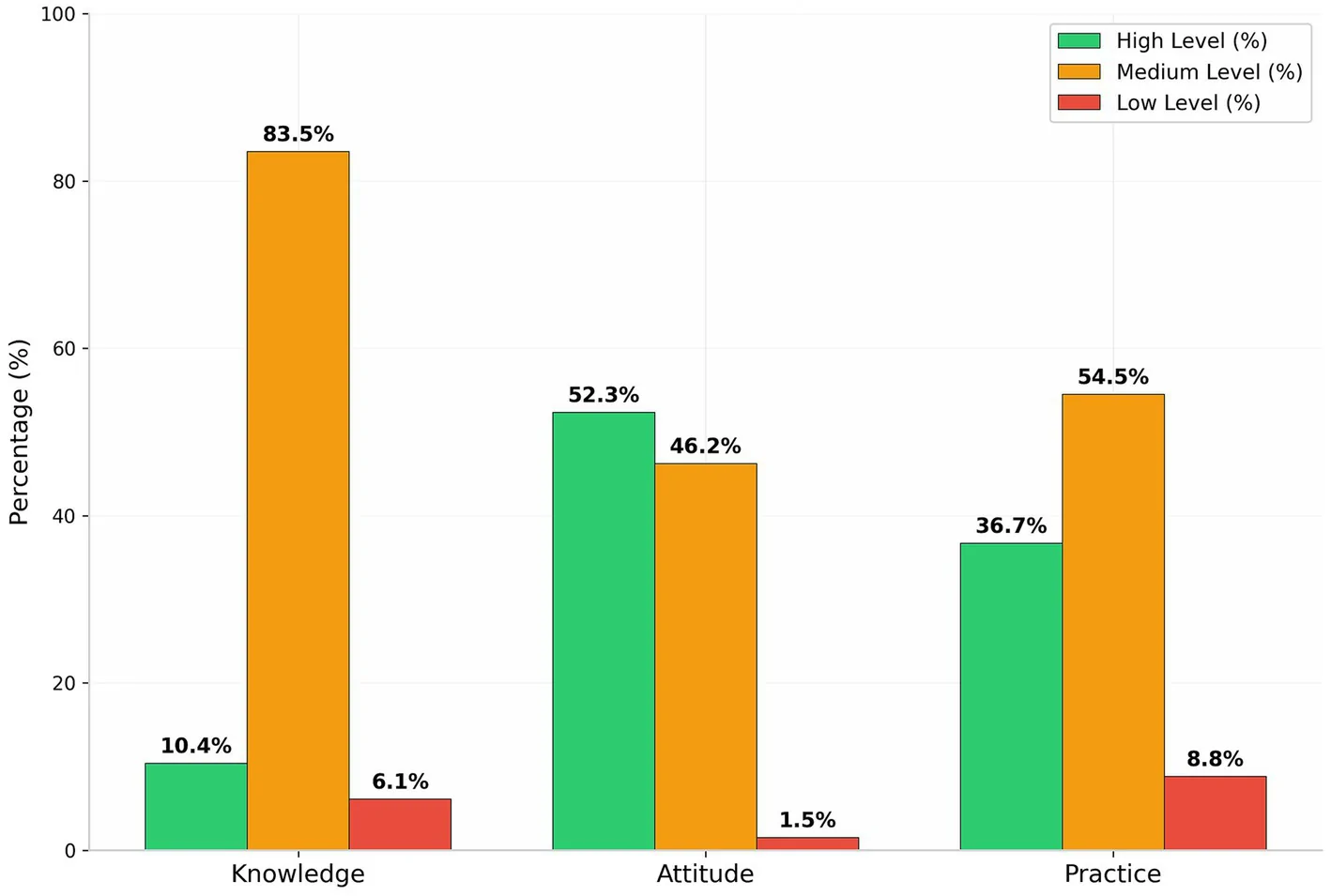
Score grade distribution of KAP on oxygen therapy among medical staff.

### 3.2 Comparison of oxygen therapy knowledge, attitude, and practice scores among healthcare workers with different characteristics

There were statistically significant differences (*p* < 0.05) in knowledge scores by gender (*t* = −3.210, *p* = 0.001) and occupation (*t* = −2.582, *p* = 0.010). Female participants and nurses had higher knowledge scores than male participants and physicians, respectively. In the regression analysis, occupation was coded as Doctor = 1 and Nurse = 2; the positive coefficient for occupation (*B* = 4.956, *p* = 0.041) indicates higher knowledge scores among nurses than among doctors, consistent with the descriptive findings. However, given the very small number of physicians (*n* = 9), this occupation-related finding should be interpreted with caution. Significant differences were also observed across education levels, professional titles, participation in training, and hospital levels for all three dimensions (see Table 3; Figure 2).

**Table 3:** Comparison of knowledge, attitude, and practices scores on oxygen therapy among healthcare workers with different characteristics.

Project	Number	Knowledge	Attitude	Practice
Gender				
Male	120	54.58 ± 9.04	25.49 ± 4.35	39.67 ± 7.88
Female	1,052	56.68 ± 6.44	25.76 ± 3.41	40.03 ± 7.36
*t*		−3.210	−0.768	−0.513
*p*		0.001	0.432	0.608
Age				
<25 years old	50	53.78 ± 8.83	24.86 ± 4.96	39.76 ± 8.51
25–30 years old	321	56.73 ± 6.31	25.72 ± 3.53	39.83 ± 7.47
31–40 years old	621	56.44 ± 6.79	25.82 ± 3.36	39.91 ± 7.20
41–50 years old	142	56.82 ± 6.64	25.91 ± 3.32	40.73 ± 7.21
>50 years old	38	56.82 ± 6.77	24.82 ± 4.14	40.00 ± 7.42
*F*		2.219	1.610	0.424
*p*		0.065	0.169	0.791
Educational qualifications				
Technical secondary school	5	46.80 ± 18.71	20.20 ± 8.04	36.80 ± 15.45
Junior college	98	56.68 ± 6.93	23.97 ± 3.99	37.36 ± 8.23
Undergraduate degree	1,050	56.57 ± 6.59	25.92 ± 3.36	40.27 ± 7.23
Master’s degree candidate	16	55.06 ± 4.74	26.93 ± 3.02	41.38 ± 6.75
Doctoral students and above	3	36.00 ± 9.00	19.33 ± 4.93	29.33 ± 2.52
*F*		9.939	13.541	5.464
*p*		<0.001	<0.001	<0.001
Occupation				
Doctor	9	50.67 ± 12.50	24.22 ± 4.63	37.78 ± 8.39
Nurse	1,163	56.51 ± 6.70	25.74 ± 3.50	40.01 ± 7.40
*t*		−2.582	−1.293	−0.901
*p*		0.010	0.196	0.368
Title				
Nurse/doctor	77	54.05 ± 8.28	24.44 ± 4.73	39.92 ± 9.10
Nurse/resident physician	367	56.72 ± 6.81	25.86 ± 3.36	39.78 ± 7.62
Senior nurse practitioner/chief physician	649	56.81 ± 6.40	25.77 ± 3.45	40.13 ± 7.03
Associate chief nurse/associate chief physician	76	55.26 ± 6.40	26.25 ± 3.01	40.28 ± 7.69
Chief nurse/chief physician	3	42.00 ± 18.73	22.33 ± 5.51	32.33 ± 5.51
*F*		7.154	3.880	0.962
*p*		<0.001	0.004	0.427
Work department				
Internal medicine	320	55.91 ± 7.05	25.85 ± 3.56	39.56 ± 7.62
Surgery	220	57.01 ± 6.26	25.75 ± 3.55	39.91 ± 7.63
Obstetrics and gynecology	63	56.84 ± 7.20	25.35 ± 3.79	39.75 ± 7.47
Pediatrics	139	55.06 ± 6.89	25.69 ± 3.32	41.19 ± 6.75
Emergency department	92	57.45 ± 7.84	25.45 ± 3.19	39.65 ± 6.63
Intensive care unit	249	57.01 ± 6.25	25.81 ± 3.55	39.95 ± 7.07
Operating room	12	48.75 ± 10.88	25.25 ± 4.05	38.83 ± 12.05
Outpatient service	39	58.52 ± 8.53	26.77 ± 3.15	42.03 ± 7.09
Hyperbaric oxygen chamber	0	–	–	–
Other	38	36.76 ± 5.77	24.63 ± 3.75	39.63 ± 8.87
*F*		4.149	1.151	1.039
*p*		<0.001	0.326	0.404
Length of service				
≤2 years	59	54.81 ± 8.05	25.24 ± 4.77	40.88 ± 8.40
3–5 years	152	56.55 ± 6.62	25.55 ± 3.57	39.07 ± 7.26
6–10 years	312	56.84 ± 6.91	25.73 ± 3.42	39.76 ± 7.52
11–15 years	384	56.29 ± 6.81	25.96 ± 3.41	39.87 ± 7.18
≥16 years	265	56.58 ± 6.52	25.62 ± 3.41	40.79 ± 7.42
*F*		1.210	0.872	1.687
*p*		0.305	0.480	0.151
Employment form				
System of contract	723	56.38 ± 6.84	25.75 ± 3.48	39.80 ± 7.20
Personnel agency system	299	56.72 ± 6.33	25.77 ± 3.42	40.62 ± 7.34
Establishment of institutions	118	56.74 ± 7.33	25.64 ± 3.72	40.53 ± 7.91
Other	32	54.91 ± 7.17	25.25 ± 4.44	36.63 ± 9.80
*F*		0.817	0.247	3.289
*p*		0.484	0.863	0.020
Have participated in oxygen therapy training				
Yes	879	56.45 ± 6.77	26.01 ± 3.43	40.64 ± 7.11
No	293	56.49 ± 6.80	24.90 ± 3.62	38.05 ± 7.94
*t*		−0.087	4.708	5.245
*p*		0.931	<0.001	<0.001
Whether oxygen therapy was provided				
Yes	1,046	56.55 ± 6.53	25.85 ± 3.44	40.20 ± 7.25
No	126	55.75 ± 8.56	24.75 ± 3.92	38.27 ± 8.44
*t*		1.241	3.326	2.775
*p*		0.215	0.001	0.006
Have you studied oxygen therapy materials				
Yes	1,019	56.55 ± 6.63	25.89 ± 3.44	40.34 ± 7.21
No	153	55.85 ± 7.64	24.69 ± 3.81	37.68 ± 8.30
*t*		1.198	3.942	4.175
*p*		0.231	<0.001	<0.001
Level of hospital				
Tertiary hospital	930	56.84 ± 6.44	25.93 ± 3.49	40.50 ± 7.27
Tertiary B hospital	176	55.28 ± 7.78	25.02 ± 3.71	38.31 ± 7.34
Second class hospital	26	53.27 ± 8.80	24.62 ± 2.95	35.31 ± 7.43
Second class B hospital	1	41.00	30.00	48.00
Class A Level 1 Hospital	0	–	–	–
Class B Level 1 Hospital	0	–	–	–
Other	39	55.36 ± 6.73	24.79 ± 2.71	38.49 ± 6.91
*F*		5.134	4.338	6.776
*p*		<0.001	0.002	<0.001

**Figure 2:**
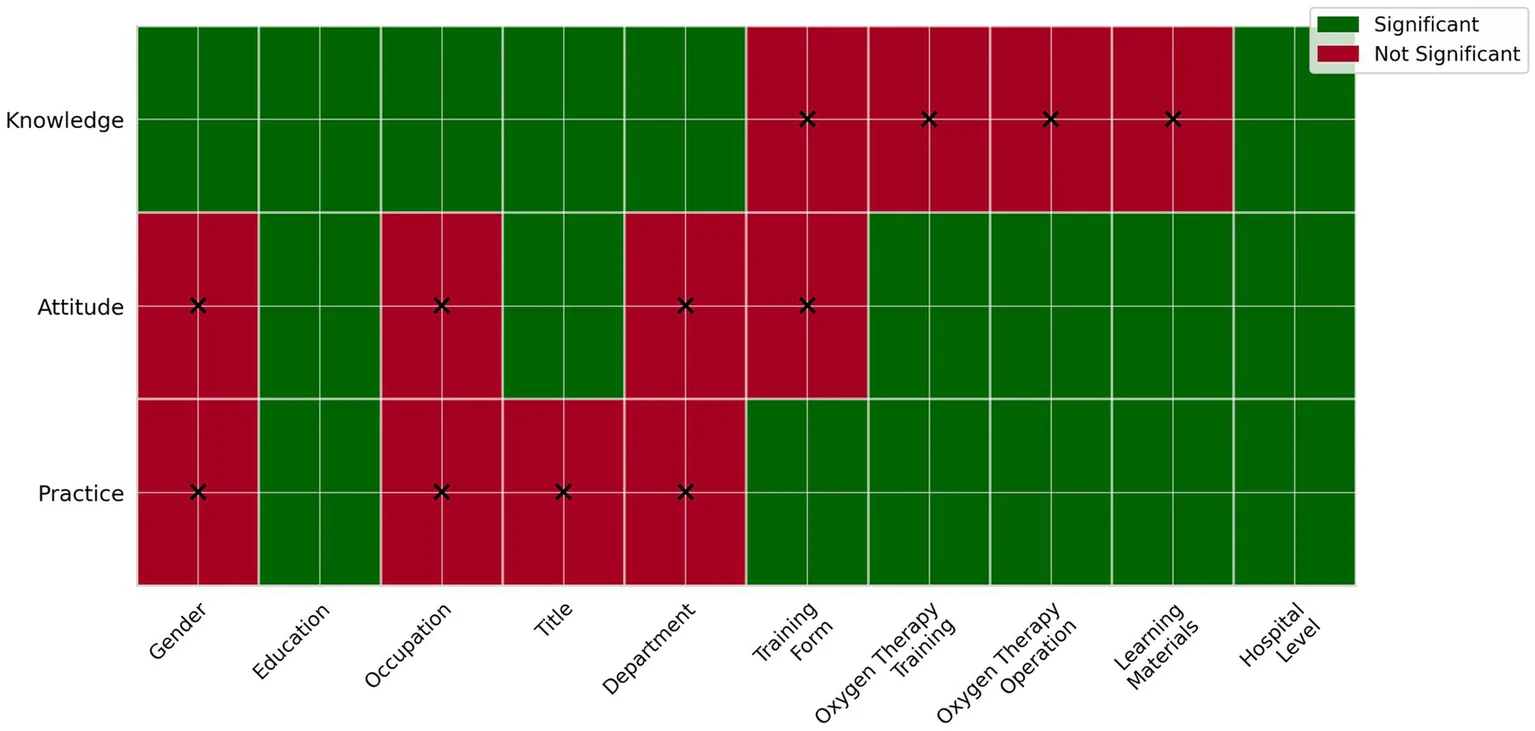
Summary of univariate analysis results.

### 3.3 Multiple linear regression analysis of factors influencing medical staff’s knowledge, attitude, and practice of oxygen therapy

For multiple linear regression analysis, variables that were statistically significant in univariate analysis were used as independent variables. For ordinal categorical variables such as job title, education level, and hospital grade, we used continuous numerical coding in the regression model, which is statistically equivalent to testing for trend as continuous variables. The results of sensitivity analysis of dummy variable coding were highly consistent with the core conclusions of the original analysis (continuous variable coding). The direction and significance of the main influencing factors such as gender, occupation, hospital level, training participation, working years and attitude scores did not change. However, for the sake of rigor, these findings should be considered exploratory. Future studies can use dummy variable coding to obtain more detailed information on comparison between groups. The coding method for the independent variables is shown in Table 4, and the results of the multiple linear regression analysis are shown in Table 5 and Figure 3.

**Table 4:** Assignment of independent variables.

The project	Mode of assignment
Gender	Male = 1; Female = 2
Occupation	Doctor = 1; Nurse = 2
Educational qualifications	College degree or below = 1; Bachelor degree = 2; Master degree or above = 3
Title	Nurse/Doctor = 1; Nurse/Resident = 2; Physician Senior Nurse Practitioner/Chief Physician = 3; Senior professional title = 4
Length of service	≤2 years = 1;3–5 years = 2; 6–10 years = 3; 11–15 years = 4; ≥16 years = 5
Level of hospital	Tertiary hospital = 1; Tertiary B hospital = 2; Second class hospital = 3; Second class B hospital = 4; Class A Level 1 Hospital = 5; Class B Level 1 Hospital = 6; Other = 7
Have participated in training	Yes = 1; No = 2

Occupation was coded as Doctor = 1 and Nurse = 2. Therefore, a positive regression coefficient for occupation indicates higher scores among nurses than among doctors.

**Table 5:** Results of multiple linear regression analysis.

Dependent variable	Independent variable	*B* value	SE	*β* value	*t* value	*p* value	95% CI
Knowledge	Constant quantity	38.095	6.384	–	5.967	<0.001	25.570–50.620
	Occupations	4.956	2.425	0.064	2.044	0.041	0.201–9.711
	Gender	2.167	0.696	0.097	3.114	0.002	0.802–3.532
	Professional title	−0.326	0.159	−0.071	−2.049	0.041	−0.638 to −0.014
	Level of hospital	−0.470	0.183	−0.079	−2.573	0.010	−0.829 to −0.111
Attitude	Constant quantity	15.784	3.265	–	4.835	<0.001	9.379–22.189
	Occupations	2.755	1.240	0.069	2.222	0.026	0.322–5.188
	Educational qualifications	1.499	0.326	0.147	4.601	<0.001	0.860–2.138
	Level of hospital	−0.272	0.093	−0.088	−2.913	0.004	−0.455 to −0.089
	Have participated in training	−0.822	0.288	−0.101	−2.853	0.004	−1.387 to −0.257
Practice	Constant quantity	12.795	6.132	–	2.087	0.037	0.765–24.825
	Attitude	1.050	0.055	0.499	19.057	<0.001	0.942–1.158
	Professional title	−0.933	0.429	−0.090	−2.174	0.030	−1.775 to −0.091
	Length of service	0.701	0.346	0.106	2.022	0.043	0.022–1.380
	Have participated in training	−1.363	0.533	−0.080	−2.558	0.011	−2.408 to −0.318

Knowledge *R*^2^ = 0.034, adjusted *R*^2^ = 0.019, *F* = 2.233, *p* = 0.002; attitude *R*^2^ = 0.060, adjusted *R*^2^ = 0.045, *F* = 4.086, *p* < 0.001; practice *R*^2^ = 0.288, adjusted *R*^2^ = 0.274, *F* = 21.003, *p* < 0.001.

**Figure 3:**
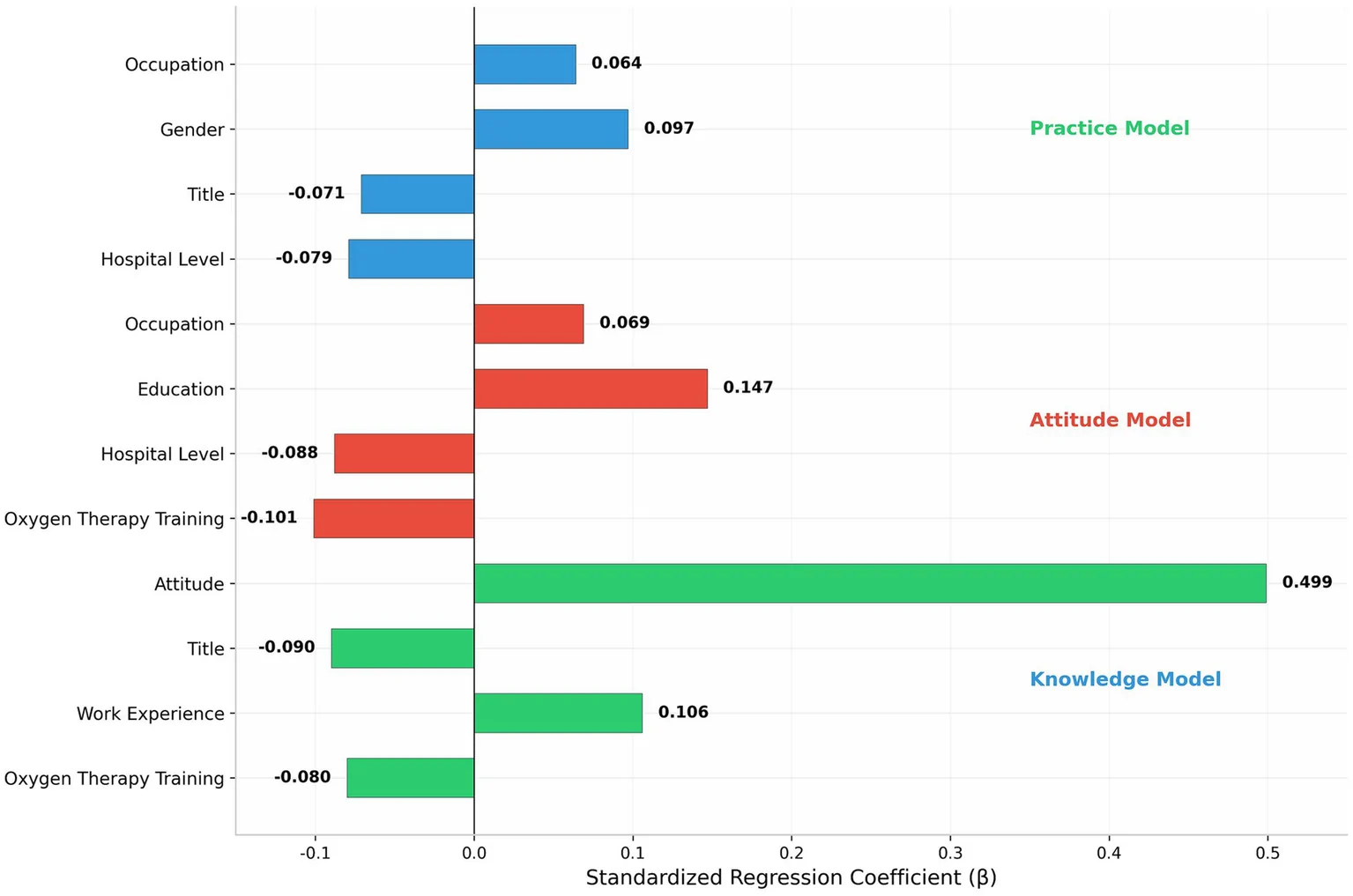
Standardized regression coefficients (*β*) of multiple linear regression models.

## 4 Discussion

### 4.1 Overall knowledge, attitudes, and practices of clinical healthcare personnel regarding oxygen therapy are at a moderate level, showing an atypical pattern of ‘best attitude, lagging knowledge’

This study shows that healthcare personnel scored highest in the attitude dimension of oxygen therapy (standard score 85.77 ± 11.71), with 52.30% at a high level; the practice dimension was next (79.99 ± 14.82); and the knowledge dimension was the lowest (75.28 ± 9.03), with 83.45% at a moderate level. This inverted pattern of ‘positive attitude, lagging knowledge’ deviates from the classic Knowledge-Attitude-Practice (KAP) model (11) (knowledge → attitude → practice), suggesting that current healthcare personnel’s practice of oxygen therapy may rely more on experiential habits rather than systematic knowledge, this is consistent with the findings of Bakare et al. (12).

Within the knowledge dimension, scores for ‘oxygen therapy indications’, oxygen therapy monitoring, and ‘HFNC-related knowledge’ were relatively high, reflecting a reasonable grasp of basic oxygen therapy skills, which is related to the widespread inclusion of oxygen therapy as a routine nursing procedure in both academic curricula and pre-job training. However, scores for ‘clinical manifestations of oxygen toxicity’ (1.54 ± 1.16), ‘preparation before oxygen therapy’ (2.73 ± 1.92), and ‘oxygen therapy humidification’ (3.23 ± 1.35) were significantly low, indicating insufficient mastery by healthcare personnel in identifying oxygen therapy complications, operational details, and new developments (such as updates in humidification standards) (13). This finding was consistent with the results of the survey of medical staff by Ke Ziwei et al. (14), Aynalem et al. (15), reflecting the systemic defects in the mechanism of oxygen therapy knowledge updating.

### 4.2 Attitude dimension: recognizes the value but lacks deep understanding, showing a tendency toward “passive agreement”

The overall score of the attitude dimension is relatively high, but the score for the item “Oxygen is a drug” is relatively low (3.76 ± 1.22). Insufficient awareness of the drug properties may result in weak awareness of prescription and precision in oxygen therapy (16), remaining at a superficial understanding of “oxygen therapy is important, actively providing oxygen,” without yet establishing a deep concept of “oxygen therapy requires precise prescription, dynamic monitoring, and prevention of side effects.” (17). Furthermore, the score for “Interested in knowledge about oxygen therapy” (4.22 ± 0.72) is lower than the score for “Recognition of the importance of oxygen therapy” (4.57 ± 0.76), showing a characteristic of passive agreement with “rational recognition but emotional indifference.” This may be related to oxygen therapy being seen as repetitive basic work with insufficient professional challenge, and may also be affected by heavy clinical workloads and limited energy for learning. Lack of interest directly restricts proactive learning practice, echoing the relatively low score for “actively accumulating knowledge” in the practice dimension (3.91 ± 0.99).

### 4.3 Practice dimension: strong awareness of safety prevention, but insufficient precision oxygen therapy and proactive learning practice

In the practice dimension, scores for ‘avoiding hypoxia’ (4.29 ± 0.84) and ‘avoiding hyperoxia’ (4.09 ± 0.94) were relatively high, indicating that medical staff have a strong awareness of safety prevention in oxygen therapy. However, the lowest score was for ‘avoiding SpO_2_ reaching 100%’ (3.62 ± 1.16), It indicates that the concept of ‘target oxygen saturation range’ is insufficient, rather than just ignorance of the extreme value of ‘SpO_2_ reaching 100%’, which reflects a lack of understanding of the guidement-recommended permissive hypoxemia or conservative oxygen therapy strategy. The 2020 “Adult Oxygen Therapy Standard” (18) and multiple studies (19, 20) emphasize that for patients without a risk of hypercapnia, the SpO_2_ target should be set at 94–98% rather than aiming for 100%. This practice shortcoming is closely related to the weak knowledge of oxygen toxicity and oxygen target setting in the knowledge dimension, further confirming the foundational role of knowledge updating in practice optimization (21).

### 4.4 Impact factor analysis: training participation was associated with attitudes and practices but lacked association with knowledge improvement

Multiple regression analysis revealed differentiated influencing factors across three dimensions:

The knowledge dimension is affected by gender, occupation, professional title, and hospital level. Those who are female, those with lower professional titles, and those in higher-level hospitals (tertiary hospitals) scored higher in knowledge. It is worth noting that in this study, the proportion of nurses was as high as 99.2%, and the sample size of doctors was only 9. In the univariate analysis, nurses had higher knowledge scores than doctors (56.51 ± 6.70 vs. 50.67 ± 12.50). In the multiple linear regression model, occupation was coded as Doctor = 1 and Nurse = 2; the positive coefficient for occupation (*B* = 4.956) therefore indicates that nurses had higher knowledge scores than doctors, which is consistent with the descriptive results. However, given the extremely small number of physicians (*n* = 9), this occupation-related finding is unstable and should be interpreted with caution. It should not be generalized to physicians, and future studies should include a larger and more balanced sample of physicians and nurses. In addition, the results of this study showed that training did not enter the knowledge regression equation, and there was no difference in knowledge scores between nurses with and without training (*p* = 0.931), which suggested that the current routine training may have reached its bottleneck in improving the basic knowledge score, that is, for nurses who have already mastered the basic operation, the existing training content is difficult to further improve their knowledge level. Especially cutting edge and updating knowledge.

The attitude dimension is influenced by occupation, educational level, hospital level, and training. The multiple regression models explained only a small proportion of variance in knowledge (adjusted *R*^2^ = 0.019) and attitude (adjusted *R*^2^ = 0.045), whereas the practice model had moderate explanatory power (adjusted *R*^2^ = 0.274). This is not consistent with the findings of Oza et al. (22) and Desalu et al. (23). This suggests that unmeasured factors—such as institutional culture, peer influence, individual learning motivation, clinical workload, and access to updated educational materials—may play larger roles in shaping nurses’ knowledge and attitudes than the demographic and training variables we assessed. Future research should incorporate these contextual factors to better understand the determinants of KAP regarding oxygen therapy.

The practice dimension is influenced by attitude, professional title, years of work experience, and training. Although training participation was not significantly associated with knowledge scores (*p* = 0.931 in univariate analysis), training participation was associated with better attitudes (*B* = −0.822, *p* = 0.004) and practices (*B* = −1.363, *p* = 0.011), consistent with the coding of training participation as Yes = 1 and No = 2. This discrepancy may reflect that current training programs emphasize operational procedures and safety awareness rather than the underlying pathophysiology and updated evidence-based guidelines (e.g., precise SpO_2_ targeting, humidification standards, recognition of oxygen toxicity). Additionally, the lack of standardized content and post-training reinforcement across hospitals may limit knowledge retention. This is consistent with findings from other studies in resource-limited settings (24, 25).

Based on the above findings, the following recommendations are proposed:

(1) Restructure training content: Shift from basic operational training to updated guidelines, complication recognition, and precise oxygen therapy target setting, incorporating participatory methods such as case teaching and scenario simulation to stimulate learning interest;
(2) Establish oxygen therapy quality control standards: Develop hospital-specific oxygen therapy protocols based on domestic and international guidelines, specifying SpO_2_ target ranges, monitoring frequency, and flow adjustment indications, achieving a shift from “experience-driven” to “standard-driven” practice;
(3) Implement hierarchical management: Strengthen basic training for personnel in lower-level hospitals and with lower professional titles, while focusing on the latest updates for more senior staff;
(4) Promote prescription-based oxygen therapy: Drawing on the “medication management” model, establish a closed-loop management system for oxygen therapy orders, execution, and monitoring, reinforcing the perception of “oxygen as a drug.”

## 5 Conclusion

This nurse-dominant study (99.2% nurses) indicates that clinical nurses in Anhui Province have moderate KAP levels regarding oxygen therapy, with relatively positive attitudes and acceptable self-reported practices, but notable knowledge deficiencies, particularly regarding complications, precise targeting, and updated standards. Gender, occupation, professional title, hospital level, and training participation were associated with KAP scores, but the occupation-related result was based on only 9 physicians and should be interpreted cautiously. Training participation was associated with better attitudes and practices but not with knowledge, suggesting that current training may need to be restructured to emphasize knowledge updating and clinical reasoning. The low explanatory power of the knowledge and attitude models highlights the need for future research to examine contextual and motivational factors. Establishing evidence-based quality control standards and implementing tiered, participatory training programs are recommended. The extremely small physician sample limits generalizability to non-nursing healthcare professionals. In addition, the sample of this study was nested in 24 hospitals, and there may be a certain clustering effect within hospitals. Because this study did not measure hospital-level characteristic variables (e.g., hospital oxygen therapy management system, quality control standards, etc.) and did not use a multilevel model for analysis, potential clustering effects may have affected the robustness of the results. Future studies should incorporate hospital-level variables and use multilevel regression models for analysis.

## Data Availability

The original contributions presented in the study are included in the article/Supplementary material, further inquiries can be directed to the corresponding author.
