# Supplementary material for: Status and influencing factors of knowledge, attitudes, and practices regarding oxygen therapy among clinical nurses in Anhui Province, China
Source: Front Public Health. 2026 Sep 28;14:1945141. doi: 10.3389/fpubh.2026.1945141 (PMC13655115; doi:10.3389/fpubh.2026.1945141)
Supplement: Supplementary file 1 [file Data_Sheet_1.PDF]

# Clinical Staff Knowledge, Attitude, and Practice Questionnaire on Oxygen Therapy

This questionnaire aims to investigate the current status of knowledge, attitude, and practice (KAP) regarding oxygen therapy among clinical healthcare professionals, and to provide a reference for developing rational oxygen therapy strategies. This survey is anonymous and your responses will be kept confidential. We sincerely appreciate you taking the time to complete this questionnaire. Please select the option that best reflects your actual situation. Thank you for your support and cooperation.

## Part I: Demographic Information

(1) Name of your hospital: ________________________________

(2) Your profession:
- Physician
- Nurse

(3) Your gender:
- Male
- Female

(4) Your age:
- <25 years
- 25–30 years
- 31–40 years
- 41–50 years
- >50 years

(5) Marital status:
- Single
- Married
- Divorced
- Remarried
- Widowed
- Other

(6) Childbearing status:
- Has children
- No children

(7) Age of your oldest child (for those who have children):
- 0–1 year
- 1–4 years

- 5–11 years
- 12–18 years
- >18 years

(8) Educational level:
- Secondary technical school
- Associate degree
- Bachelor's degree
- Master's degree
- Doctoral degree or above

(9) Professional title:
- Nurse/Resident Physician
- Senior Nurse/Attending Physician
- Supervisor Nurse/Associate Chief Physician
- Associate Chief Nurse/Chief Physician
- Chief Nurse/Chief Physician

(10) Administrative position:
- None
- Preceptor
- Head Preceptor
- Department Secretary
- Chief Resident
- Head Nurse/Associate Head Nurse
- Department Director/Associate Director

(11) Are you a certified specialist nurse?
- No
- Respiratory Therapist
- Critical Care Specialist Nurse
- Pediatric Specialist Nurse
- Emergency Specialist Nurse
- Other Specialist Nurse

(12) Employment type:
- Contract-based
- Personnel agency system
- Permanent staff (government-sponsored)
- Other

(13) Years of clinical experience:
- ≤2 years
- 3–5 years

- 6–10 years
- 11–15 years
- ≥16 years

(14) Type of hospital:
- General hospital
- Specialty hospital
- Private hospital
- Community hospital

(15) Hospital tier:
- Tertiary Grade A
- Tertiary Grade B
- Secondary Grade A
- Secondary Grade B
- Primary Grade A
- Primary Grade B
- Other

(16) Current working department:
- Internal Medicine
- Surgery
- Obstetrics and Gynecology
- Pediatrics
- Emergency Department
- Intensive Care Unit
- Operating Room
- Outpatient Department
- Hyperbaric Oxygen Department
- Other

(17) Departments in which you have worked for more than one month (select all that apply):
- None
- Emergency Department
- Intensive Care Unit
- Operating Room
- Respiratory Department
- Hyperbaric Oxygen Department
- Other

(18) Have you ever participated in oxygen therapy-related training?
- Yes
- No

(19) Have you ever provided oxygen therapy to patients?
- Yes
- No

(20) Have you ever studied oxygen therapy-related materials?
- Yes
- No

(21) Sources of your knowledge on clinical oxygen therapy (select all that apply):
- Departmental academic activities
- Relevant books and literature
- Academic conferences and lectures
- Self-study due to personal interest or work needs
- Training courses
- Never paid attention to this area
- Other (please specify): ________

Part II: Knowledge Section on Clinical Oxygen Therapy

(1) What is the purpose of oxygen therapy?
- a. To increase arterial partial pressure of oxygen ($PaO_2$) and oxygen saturation ($SpO_2$), correct hypoxemia, and alleviate tissue hypoxia.
- b. To relieve symptoms such as dyspnea.
- c. To improve the patient's quality of life and exercise tolerance.
- d. To protect the function of vital organs (e.g., heart, brain, kidneys) and prevent or reduce complications.
- e. All of the above.

Answer: e

(2) What are the indications for oxygen therapy?
- a. Respiratory failure of various causes, including Type I respiratory failure ($PaO_2 < 60$ mmHg with low or normal $PaCO_2$) and Type II respiratory failure ($PaO_2 < 60$ mmHg with $PaCO_2 > 50$ mmHg).
- b. Cardiovascular diseases such as acute myocardial infarction and heart failure that lead to reduced cardiac output and tissue hypoxia.
- c. Conditions such as severe anemia or carbon monoxide poisoning that reduce hemoglobin oxygen-carrying capacity or impair oxygen utilization.
- d. Postoperative patients, especially after major thoracic or abdominal surgery, for the prevention and treatment of postoperative hypoxemia.
- e. All of the above.

Answer: e

(3) Which of the following is NOT a major clinical manifestation of hypoxia?
- a. Restlessness and tachycardia
- b. Wheezing and nasal flaring
- c. Peripheral cyanosis
- d. Hypotension
- e. Altered mental status

Answer: d

(4) Which of the following is NOT a clinical manifestation of oxygen toxicity?
- a. Elevated body temperature
- b. Nausea
- c. Restlessness
- d. Substernal discomfort
- e. Tachypnea

Answer: a

(5) Which of the following descriptions regarding preparation before oxygen therapy administration is INCORRECT?
- a. Prepare oxygen delivery equipment (oxygen cylinder or central oxygen supply), humidification device, oxygen delivery device, and related supplies according to the prescription and environmental conditions. When using an oxygen cylinder, connect the pressure gauge first, open the valve, and ensure the cylinder pressure is > 0.2 MPa.
- b. Verify patient information, oxygen therapy duration, delivery method, and flow rate.
- c. Instruct the patient to assume a comfortable position and explain the purpose, method, and precautions of oxygen therapy.
- d. Confirm that the flow meter, oxygen delivery device (including tubing), and humidification device are securely connected.
- e. Adjust the oxygen flow rate with the flow meter float at the center position and check device patency.

Answer: a

(6) Which of the following descriptions regarding oxygen delivery methods is INCORRECT?
- a. For oxygen flow requirements of 1–5 L/min, nasal cannula is recommended.
- b. For oxygen flow requirements of 5–10 L/min with risk of hypercapnia, simple face mask is recommended.
- c. For oxygen flow requirements of 6–15 L/min without risk of hypercapnia, reservoir mask is recommended.
- d. For oxygen flow requirements of 2–15 L/min with risk of hypercapnia, Venturi mask is recommended.
- e. For oxygen flow requirements of 8–80 L/min with pH > 7.3, high-flow nasal cannula (HFNC) oxygen therapy may be selected, especially when flow requirement exceeds 15 L/min.

Answer: b

(7) What is the recommended oxygen delivery method for patients with severe simple hypoxemia?
- a. Nasal cannula at 3 L/min
- b. Simple face mask at 6 L/min
- c. Reservoir mask at 8 L/min
- d. Venturi mask at 10 L/min
- e. High-flow nasal cannula device

Answer: b

(8) Which of the following descriptions regarding the application of oxygen delivery devices is INCORRECT?
- a. For nasal cannula, place the tip into the patient's nostrils to a depth of 1.5 cm.
- b. For simple face mask, place it over the patient's face, position the straps behind the head with appropriate tightness, and ensure good facial seal.
- c. For reservoir mask, before connecting to the patient, check the one-way valve function, adjust the oxygen flow, and inflate the reservoir bag. Place the mask over the face with straps behind the head at appropriate tightness. Keep the reservoir bag inflated during use.
- d. For Venturi mask, place it over the patient's face with straps behind the head at appropriate tightness and ensure good facial seal.
- e. Adjust the oxygen flow first, then set the oxygen concentration, ensuring that the flow rate matches the Venturi device markings.

Answer: e

(9) Which of the following is correct regarding oxygen therapy for COPD patients?
- a. A target $SpO_2$ range should be prescribed, with a recommended target of 88%–92%.
- b. $SpO_2$ should be kept below 96% to prevent oxygen toxicity and $CO_2$ retention caused by high-flow oxygen therapy.
- c. Nasal cannula or face mask oxygen therapy is recommended for 15–24 hours per day, starting at 1 L/min with flow adjustments at 30-minute intervals, increasing by 1 L/min until $SpO_2 \geq 90\%$ or $PaO_2 \geq 60$ mmHg.
- d. If $SpO_2$ cannot be maintained at 88%–92%, continuous oxygen therapy of at least 15–20 hours per day should be provided with appropriate documentation.
- e. All of the above.

Answer: e

(10) Which of the following statements regarding the prevention and management of hypercapnia is correct?
- a. For patients at risk of hypercapnia, controlled oxygen therapy should be administered with enhanced airway management to maintain airway patency.
- b. If the patient shows decreased $SpO_2$, altered mental status, tachypnea progressing to

bradypnea, tachycardia or bradycardia, or decreased urine output, hypercapnia should be suspected and arterial blood gas analysis should be ordered.
- c. Oxygen therapy should be adjusted under the guidance of arterial blood gas analysis to maintain the target $SpO_2$ while closely monitoring $PaCO_2$ changes.
- d. When necessary, respiratory stimulants or mechanical ventilation should be administered as prescribed to increase ventilation and correct hypercapnia.
- e. All of the above.

Answer: e

(11) Which of the following descriptions regarding the advantages and disadvantages of nasal cannula oxygen therapy is INCORRECT?
- a. Simple, rapid, and cost-effective, meeting the needs of most mild cases.
- b. Relatively well-tolerated; does not interfere with eating or speaking.
- c. Delivers a stable $FiO_2$ that is not affected by tidal volume or respiratory rate.
- d. Cannot deliver high concentrations of oxygen.
- e. Insufficient humidification with prolonged use or flow rates above 5 L/min, leading to poor tolerance.

Answer: c

(12) Which of the following descriptions regarding the advantages and disadvantages of Venturi mask oxygen therapy for patients with hypoxemia and hypercapnia is INCORRECT?
- a. Allows precise oxygen delivery.
- b. The patient's breathing pattern may affect the delivered $FiO_2$.
- c. Essentially no $CO_2$ rebreathing.
- d. Higher cost and limited humidification efficacy with limited $FiO_2$ range.
- e. Oxygen flow rate must be matched with the delivered $FiO_2$.

Answer: b

(13) Which of the following statements regarding high-flow nasal cannula (HFNC) oxygen therapy is correct?
- a. The humidification water used in HFNC should be sterile distilled water or sterile water for injection.
- b. The recommended sequence is: power on, set parameters, apply nasal cannula, then deliver gas flow to minimize aerosol generation.
- c. The nasal cannula should be appropriately sized, and a fixation strap should ensure a secure connection to maximize the physiological dead-space flushing effect and respiratory support while minimizing unnecessary aerosolization.
- d. If hypoxemia does not improve within 1–2 hours of HFNC therapy, reassessment should be performed and alternative oxygen therapy or ventilatory support should be considered. Once hypoxemia is corrected and stabilized, flow and $FiO_2$ may be gradually reduced and HFNC may be discontinued after comprehensive evaluation.

- e. All of the above.

Answer: e

(14) Which of the following descriptions regarding humidification during oxygen therapy is INCORRECT?
- a. Humidification should be provided when oxygen flow rate > 4 L/min, or in dry environments, or when sputum is excessive, thick, and difficult to expectorate, or when patients complain of upper airway dryness even at flow rates < 4 L/min.
- b. Heated humidification is recommended for oxygen flow rates < 15 L/min, HFNC oxygen therapy, and oxygen therapy via artificial airways such as endotracheal tubes or tracheostomies.
- c. Humidification solutions should be sterile distilled water or sterile water for injection, with strict aseptic technique.
- d. Surface humidification devices may be used; submerged humidification devices may also be used.
- e. Disposable humidification devices should be preferred. For reusable humidification devices, the cleaning, disinfection, and replacement of solutions and humidification bottles should follow WS/T 510-2016 guidelines.

Answer: b

(15) What should be observed and monitored during oxygen therapy?
- a. Assess the patient's level of consciousness, heart rate, respiratory status, improvement of cyanosis, and oxygen therapy-related complications.
- b. Assess nasal mucosa; if dryness is noted, water-based lubricants should be applied.
- c. Check the connection of tubing and patient interface; damaged, broken, or visibly contaminated tubing should be replaced immediately; any accumulated fluid in HFNC circuits should be removed promptly.
- d. Evaluate $SpO_2$ or arterial blood gas results; if the target $SpO_2$ range is not achieved, or if clinical presentation or arterial blood gas results do not improve or worsen, the physician should be notified promptly.
- e. All of the above.

Answer: e

---

Part III: Attitude Section on Clinical Oxygen Therapy

Each item is rated on a 5-point Likert scale: 1 = Strongly Disagree, 2 = Disagree, 3 = Neutral, 4 = Agree, 5 = Strongly Agree.

(1) Oxygen therapy plays an important role in the management of critically ill patients.

- Strongly Disagree / Disagree / Neutral / Agree / Strongly Agree

(2) Oxygen is a drug (Note: A drug is defined as a substance that can alter or ascertain physiological functions and pathological states, and can be used for the prevention, diagnosis, and treatment of diseases).

- Strongly Disagree / Disagree / Neutral / Agree / Strongly Agree

(3) Do you think individualized oxygen therapy goals should be established for patients receiving oxygen therapy?

- Strongly Disagree / Disagree / Neutral / Agree / Strongly Agree

(4) Do you think the inhaled oxygen flow and concentration should be precisely adjusted according to the oxygen therapy goals?

- Strongly Disagree / Disagree / Neutral / Agree / Strongly Agree

(5) Are you interested in clinical oxygen therapy knowledge?

- Strongly Disagree / Disagree / Neutral / Agree / Strongly Agree

(6) Do you think you should actively learn clinical oxygen therapy-related knowledge?

- Strongly Disagree / Disagree / Neutral / Agree / Strongly Agree

Part IV: Practice Section on Clinical Oxygen Therapy

This dimension uses a 5-point Likert scale: 1 = Never, 2 = Rarely, 3 = Unsure, 4 = Often, 5 = Always.

(1) During oxygen therapy administration, do you consider the side effects of oxygen therapy?

- Never / Rarely / Unsure / Often / Always

(2) In your daily work, do you try to prevent patients from developing hypoxia? (Note: Hypoxia refers to a pathophysiological state in which oxygen supply is insufficient to meet the body's oxygen demand.)

- Never / Rarely / Unsure / Often / Always

(3) In your daily work, do you try to prevent patients from developing hyperoxia? (Note: Hyperoxia refers to oxygen administration far exceeding the body's physiological needs, including

prolonged inhalation of high-concentration oxygen [$FiO_2 \geq 50\%$] and subsequent hyperoxemia.)

- Never / Rarely / Unsure / Often / Always

(4) In your daily work, do you establish (or suggest that physicians establish) individualized oxygen therapy goals based on the patient's condition?

- Never / Rarely / Unsure / Often / Always

(5) In your daily work, do you (or suggest that physicians) precisely adjust the inhaled oxygen flow and concentration according to the oxygen therapy goals?

- Never / Rarely / Unsure / Often / Always

(6) In your daily work, do you (or suggest that physicians) use the lowest oxygen flow/concentration to maintain the patient's $SpO_2$ within the target range?

- Never / Rarely / Unsure / Often / Always

(7) For patients without risk factors for $CO_2$ retention, do you (or suggest that physicians) try to avoid $SpO_2$ reaching 100% during oxygen therapy?

- Never / Rarely / Unsure / Often / Always

(8) When a patient's oxygen concentration/flow is high but arterial oxygen saturation remains low, do you (or suggest that physicians) assess tissue oxygenation status?

- Never / Rarely / Unsure / Often / Always

(9) When arterial blood gas analysis shows $PaO_2 > 120$ mmHg, do you (or suggest that physicians) adjust the oxygen concentration/flow accordingly?

- Never / Rarely / Unsure / Often / Always

(10) In your daily work, do you actively accumulate clinical oxygen therapy-related knowledge?

- Never / Rarely / Unsure / Often / Always

What do you think should be noted in clinical oxygen therapy nursing care?

____________________________________________________

Do you have any suggestions or opinions on clinical oxygen therapy management?

______________________________________________

Thank you for your participation!
